# DNA recognition by an RNA-guided bacterial Argonaute

**DOI:** 10.1371/journal.pone.0177097

**Published:** 2017-05-17

**Authors:** Kevin W. Doxzen, Jennifer A. Doudna

**Affiliations:** 1 Biophysics Graduate Group, University of California, Berkeley, California, United States of America; 2 Department of Molecular and Cell Biology, University of California, Berkeley, California, United States of America; 3 Physical Biosciences Division, Lawrence Berkeley National Laboratory, Berkeley, California, United States of America; 4 Center for RNA Systems Biology, University of California, Berkeley, California, United States of America; 5 Howard Hughes Medical Institute, University of California, Berkeley, California, United States of America; 6 Innovative Genomics Institute, University of California, Berkeley, California, United States of America; 7 Department of Chemistry, University of California, Berkeley, California, United States of America; University of Leeds, UNITED KINGDOM

## Abstract

Argonaute (Ago) proteins are widespread in prokaryotes and eukaryotes and share a four-domain architecture capable of RNA- or DNA-guided nucleic acid recognition. Previous studies identified a prokaryotic Argonaute protein from the eubacterium *Marinitoga piezophila* (MpAgo), which binds preferentially to 5′-hydroxylated guide RNAs and cleaves single-stranded RNA (ssRNA) and DNA (ssDNA) targets. Here we present a 3.2 Å resolution crystal structure of MpAgo bound to a 21-nucleotide RNA guide and a complementary 21-nucleotide ssDNA substrate. Comparison of this ternary complex to other target-bound Argonaute structures reveals a unique orientation of the N-terminal domain, resulting in a straight helical axis of the entire RNA-DNA heteroduplex through the central cleft of the protein. Additionally, mismatches introduced into the heteroduplex reduce MpAgo cleavage efficiency with a symmetric profile centered around the middle of the helix. This pattern differs from the canonical mismatch tolerance of other Argonautes, which display decreased cleavage efficiency for substrates bearing sequence mismatches to the 5′ region of the guide strand. This structural analysis of MpAgo bound to a hybrid helix advances our understanding of the diversity of target recognition mechanisms by Argonaute proteins.

## Introduction

Argonaute (Ago) proteins exist in all three domains of life [1,2]. In eukaryotes, Argonautes are the core component of the RNA interference (RNAi) effector complex. RNAi utilizes RNA-guided messenger RNA (mRNA) binding to regulate gene expression at the transcriptional, post-transcriptional and translational levels [3–5]. Ago proteins form the RNA-induced silencing complex (RISC) of the RNAi pathway by binding to 20–30 nt microRNAs (miRNAs) or small-interfering RNAs (siRNAs) for silencing of mRNAs. This form of post-transcriptional gene regulation occurs either by Ago-catalyzed cleavage of targeted transcripts or by translational silencing through the recruitment of proteins for deadenylation and mRNA decay [6]. In prokaryotes, Argonautes can be RNA- or DNA-guided, and their functions have been more difficult to determine. Recent studies suggest that prokaryotic Argonautes may function as a novel form of host-defense through cleavage of foreign genetic elements [7,8]. An Argonaute protein from the alphaproteobacterium *Rhodobacter sphaeroides* (RsAgo) is a catalytically inactive Ago that associates with small endogenous RNAs, theorized to derive from mRNA degradation products, for targeting of plasmid and transposon DNA [9]. Additionally, when expressed in *E*. *coli*, the Argonaute from the eubacterium *Thermus thermophilus* (TtAgo) associates with small DNA guides and can cleave both RNA and DNA targets [10]. Similarly, the Argonaute from the archaeon *Pyrococcus furiosus* (PfAgo) binds small DNA guides, but unlike TtAgo can only cleave DNA targets [11]. Unlike other Argonautes, the Ago protein from the eubacterium *Marinitoga piezophila* (MpAgo) has been shown to preferentially bind 5′-hydroxylated guide RNAs to target ssDNA [12]. This unique preference adds to the question of how prokaryotic Argonaute guide sequences are generated and what structural features dictate guide and target binding specificity.

Crystal structures have provided substantial insights into the mode of action of Argonaute proteins. Structures of Ago proteins from *Pyrococcus furiosus* and the eubacterium *Aquifex aeolicus* revealed a conserved bilobed architecture [13,14]. The N-terminal and PIWI-Argonaute-Zwille (PAZ) domains constitute the amino-terminal lobe (N-lobe), while the middle (MID) and the catalytic RNase H-like P element-induced wimpy testis (PIWI) domains form the carboxyl-terminal lobe (C-lobe). The bacterial Argonaute from *Thermus thermophilus* (TtAgo) offered the first guide- and target-bound structures, providing mechanistic insights into guide stabilization and target binding [15–18]. The 5′ terminal nucleotide does not base-pair with the target strand, but instead is anchored within a MID domain binding pocket. Crystal structures of human Ago2 (hAgo2) revealed conserved structural similarities with prokaryotic Argonautes and added additional understanding towards the mechanism of target recognition [19–21]. A charged binding channel between the two Ago lobes preorders the seed sequence (nucleotides 2–8) of the guide into an A-form-like geometry [19,20,22]. This region of the guide acts as the primary target recognition site, although nucleotides within the 3′ region (nucleotides 13–16) of the guide also contribute to target binding [23–27]. Following seed sequence recognition, base-pairing propagates towards the 3′ end of the guide, releasing the loosely bound 3′ end from the PAZ domain [28–30]. The crystal structure of RsAgo bound to an RNA-DNA heteroduplex revealed how Argonaute proteins discriminate between nucleic acid type through the duplex structure of the seed sequence, and how the N-terminal domain assists in hybrid duplex stabilization [31]. Since the N-lobe interacts with 3′ end of the duplex and is the most divergent region of Argonaute proteins, crystallization of additional Ago ternary complexes with longer duplexes is necessary to understand the diverse mechanisms of target recognition.

Here we present a crystal structure of MpAgo bound to an RNA guide sequence and a complementary DNA target strand, providing insight into preferential targeting of ssDNA. When bound to an RNA-DNA heteroduplex, the N-terminal domain of MpAgo adopts a unique orientation, resulting in a linear conformation of the hybrid helix. The B-form heteroduplex positions the phosphate backbone of the DNA target to interact with charged residues within the N-lobe of MpAgo. Additionally, MpAgo displays a symmetric tolerance for guide-target mismatches across the helix. Our structural and biochemical findings provide insight into the diversity of mechanisms of target recognition by Argonaute proteins.

## Results

### Structural overview of MpAgo-RNA-DNA ternary complex

**Fig 1:**
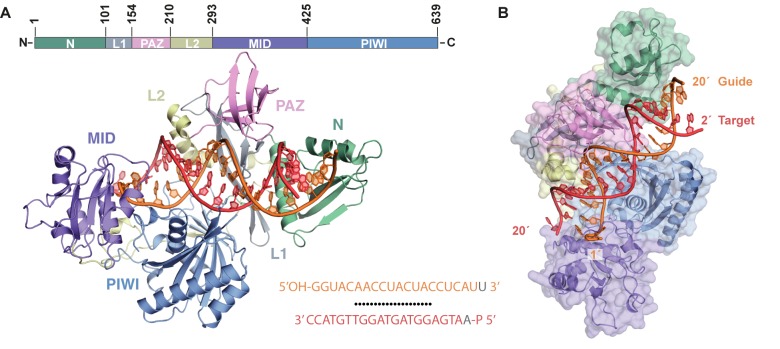
Crystal Structure of MpAgo bound to a RNA guide and DNA target heteroduplex. (A) Domain order of MpAgo with residue number demarcation. A cartoon representation of the MpAgo crystal structure with labeled N-terminal (green), Linker L1 (grey), PAZ (pink), Linker L2 (yellow), MID (purple), and PIWI (blue) domains bound to a 5′-hydroxylated 21 nt guide RNA (orange) and 5′-phosphorylated 21 nt target DNA (red). The sequence of the guide and target are aligned with black dots representing Watson-Crick base-pairing and unmodeled nucleotides are colored grey. (B) Surface representation of MpAgo with the guide RNA (orange) and target DNA (red) heteroduplex bound in-between the N- and C- lobes.

Biochemical experiments showed that MpAgo-guide RNA complexes have faster cleavage kinetics with ssDNA versus ssRNA substrates [12]. To investigate the structural basis for ssDNA recognition and cleavage, we crystallized MpAgo bound to a 5′-hydroxylated 21-nucleotide RNA guide and a complementary 5′-phosphorylated 21-nucleotide DNA target to a resolution of 3.2 Å (PDB ID: 5UX0) (Fig 1A and Table 1). In order to capture a target-bound structure, we mutated an aspartate residue to an alanine (D516A) in the enzyme’s catalytic pocket to prevent DNA cleavage. The resulting structure revealed a conserved bi-lobed architecture formed by the N-terminal (green), PAZ (pink), MID (purple), and PIWI (blue) domains, and Linkers L1 (grey) and L2 (yellow) [8]. Nucleotides 1–20 of the guide RNA (orange) and 2–21 of the target DNA (red) are ordered with a straight helical axis passing through the central cleft of the protein (Fig 1B). The heteroduplex bound by MpAgo contains more ordered nucleotides, with 20 base pairs modeled within the duplex, than previously crystallized Ago complexes (S1 Fig) [31].

**Table 1:** Data collection and refinement statistics of MpAgo ternary complex

	MpAgo:RNA:DNA
Data collection	
Space group	*P* 2_1_ 2_1_ 2_1_
Cell dimensions	
a, b, c (Å)	85.00, 130.99, 171.47
α, β, γ (°)	90, 90, 90
Resolution (Å)	47.43–3.20 (3.31–3.2)*
*R*_merge_ (%)	13.5 (93.7)
*R*_meas_ (%)	15.1 (105.3)
*I*/σ	12.04 (1.72)
CC_1/2_	99.6 (62.8)
Completeness (%)	100 (98.0)
Redundancy	4.8 (4.9)
Refinement	
Resolution (Å)	47.43–3.20
No. reflections	32,314 (3117)
*R*_work_/*R*_free_	21.6/25.8
No. atoms	
Protein	10,699
RNA/DNA	1,539
Average B-factors (Å^2^)	
Protein	63.5
RNA/DNA	111.0
R.m.s deviations	
Bond lengths (Å)	0.003
Bond angles (°)	0.69
Ramachandran	
Favored (%)	97.0
Allowed (%)	2.4
Outliers (%)	0.16

*Highest resolution shell is shown in parenthesis.

**Fig 2:**
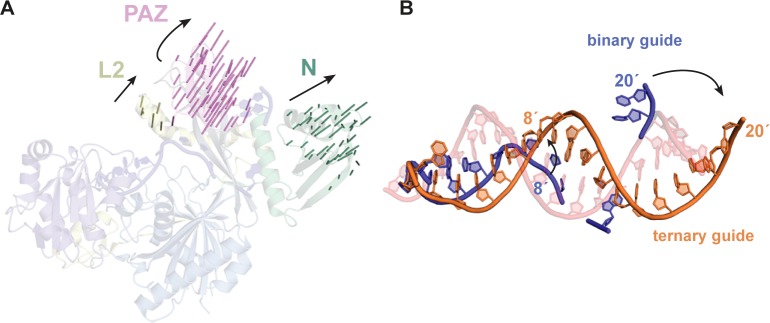
Conformational changes between MpAgo binary and ternary complexes. (A) A transparent cartoon representation of MpAgo bound to guide RNA only (PDB ID: 5I4A) with vector arrows, generated using PyMol, indicating conformational changes of MpAgo upon target binding. Black arrows represent the vector direction of the Linker L2, PAZ domain, and N domain away from the C-lobe. (B) The guide RNA (blue) from the MpAgo binary complex is overlaid with the guide RNA (orange) from the MpAgo ternary complex after alignment of the PIWI domains from the two structures. The black arrows show direction of conformational changes of the guide RNA upon target DNA (transparent red) binding.

Comparing the ternary MpAgo complex to the previously crystallized binary complex reveals prominent conformational changes [12]. Recognition and subsequent binding of the DNA target results in movement of the N-lobe away from the C-lobe to accommodate the hybrid helix (Fig 2A). Within the MpAgo-RNA complex, the 5′ end of the guide RNA is anchored into the MID domain, while the 3′ end is bound to the PAZ domain. The MpAgo ternary complex shows that the 5′ nucleotide of the guide remains bound to the MID domain and unpaired to the 3′ terminal nucleotide C21 of the DNA target (Fig 2B). The insertion of F410 between C20 and C21 disrupts the helical base stacking, splaying the terminal base away from the hybrid helix, while K279 of Linker L2 interacts with the phosphate backbone to stabilize the contorted 3′ end of the DNA target. A similar disruption at the 3′ end of the target strand by an aromatic residue is seen in both the RsAgo and TtAgo ternary complex structures (S2 Fig) [18,31]. In contrast to the 5′ end of the guide, the 3′ end is released from the PAZ domain to enable binding to the 5′ end of the target. The unique kink that was identified in the MpAgo-RNA complex is no longer seen in the hybrid helix [12].

The PIWI domain of Argonaute proteins contains a conserved DEDX catalytic tetrad (where X can be His, Asp, or Asn) [22]. In the guide-bound state, the glutamate residue (called a glutamate finger) is positioned in a flexible loop between ß-strand 3 and α-helix 1 of the PIWI domain. Upon target binding, a conformational change repositions the glutamate finger to complete the catalytic tetrad for subsequent target cleavage. In the pre-cleavage state, ß-strands 1 and 2 of the PIWI domain block the entry of the glutamate into the catalytic site. The MpAgo ternary structure shows that ß-strands 1 and 2 twist away from ß-strand 3 to create a path for the glutamate finger to complete the catalytic tetrad (S3 Fig).

The long length of this heteroduplex revealed that the 5′ region of the target DNA (positions 5–8, counting from the 5’ end) is stabilized by a positively charged groove between the N-terminal and PAZ domains (S4 Fig). The geometry of the heteroduplex within the MpAgo ternary complex aligns more closely with a perfect B-form helix instead of an A-form helix (S5 Fig). With a modeled A-form helix, the target strand is no longer positioned near the charged cleft between N-terminal and PAZ domains. The formation of the charged groove within the N-lobe is established by a unique repositioning of the N-terminal domain closer to the PAZ domain. This N-lobe arrangement allows a unique, straight orientation of the hybrid helix not seen in other Argonaute ternary complexes.

### N-terminal domain stabilizes linear conformation of RNA-DNA heteroduplex

**Fig 3:**
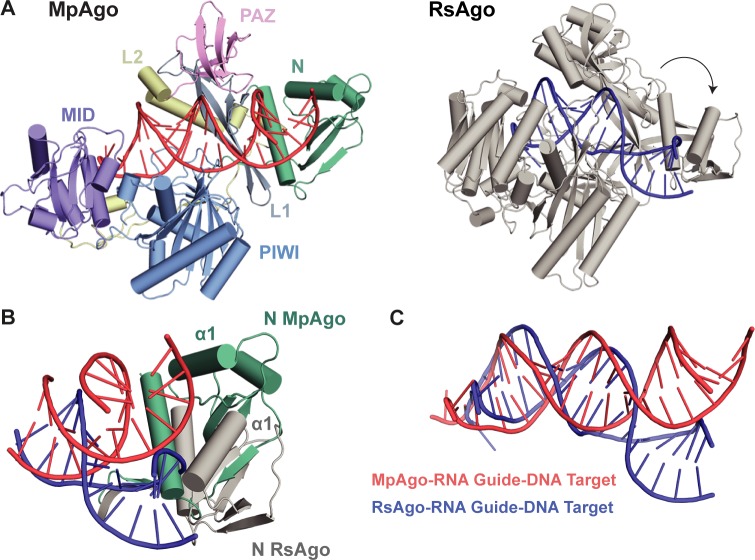
The N-terminal domain orientation of MpAgo stabilizes the RNA-DNA heteroduplex in a linear conformation. (A) MpAgo (colored by domain) was aligned to RsAgo (PDB ID: 5AWH, grey) relative to their PIWI domains. The MpAgo RNA guide (orange) and DNA target (red) heteroduplex remains in a linear conformation between the two MpAgo lobes. After two helical turns, the RNA guide (blue) and DNA target (blue) of RsAgo angles behind the PIWI domain and away from the PAZ domain. (B) The Argonaute structures from Fig 1A were superimposed and cropped to focus on the N-terminal domains and helices. The unique orientation of the MpAgo N-terminal domain (green) close to the PAZ domain corresponds with the linear conformation of the heteroduplex (red), while the angled N-terminal domain of RsAgo (grey) appears to bend the heteroduplex behind the PIWI domain. α-helix 1 of each domain is labeled to highlight the dramatic change in orientation relative to α-helix 3. (C) A cartoon representation of the linear heteroduplex of MpAgo (red) and the bent heteroduplex of RsAgo (blue), which occurs after the second helical turn.

The Argonaute protein from *Rhodobacter sphaeroides* (RsAgo) displays a preference for RNA guides and DNA substrates similar to MpAgo [31]. Comparison of our MpAgo ternary complex to the crystal structure of RsAgo bound to a hybrid helix reveals differences in both the orientation of the N-lobe and the trajectories of their guide-substrate heteroduplexes. Aligning MpAgo (colored by domain) and RsAgo (grey) relative to their PIWI domains, the most structurally conserved domain of Argonaute proteins, shows analogous positioning of the MID and PAZ domains, and Linkers L1 and L2 (Fig 3A). In contrast, the N-terminal domains show differing positions relative to the PAZ and PIWI domains between MpAgo and RsAgo. The N-terminal domain of RsAgo is positioned further from the PAZ domain and towards the PIWI domain, while the N-terminal domain of MpAgo remains proximal to Linker L1 and the PAZ domain. Specifically, the N-terminal domains show dissimilar conformations where α-helices 1 are rotated >45° relative to α-helix 3 (Fig 3B). The N-terminal domain of MpAgo is positioned close to the PAZ domain through the π-stacking of F109 of Linker L1 and F96 of the N-terminal domain (S6 Fig). Although other Ago proteins contain a conserved, aromatic residue in the F109 position, only MpAgo and the other two 5′-hydroxylated guide RNA binding Argonautes also contain an aromatic residue at position 96 for π-stacking (S7 Fig). The hydrogen bonding of N38 and D98 also helps stabilize the orientation of the N-terminal domain close to the PAZ domain. The position of α-helix 1 of the RsAgo N-terminal domain is closer to the PIWI domain through π-stacking of F55 and W85 (S6 Fig). A similar structural difference in the N-terminal domains can be seen when MpAgo is aligned to TtAgo bound to a DNA guide and DNA substrate (S8 Fig).

Our ternary structure also revealed a unique trajectory of the RNA-DNA helix within the central cleft of MpAgo. Other target-bound Argonaute structures, TtAgo and hAgo2, have shown that the N-terminal domain can act as a wedge and promote duplex unwinding [32]. In contrast, the N-terminal domain of MpAgo does not split the guide and target strands, but instead the helix remains intact through the central channel of the protein. The 5′ region of the MpAgo DNA target (positions 5–8) interacts through the phosphate backbone with charged residues (K93, K107, and K174) positioned at the interface of Linker L1 and the PAZ and N-terminal domains (S9 Fig). Although a positively charged residue is conserved amongst Argonaute proteins at position 93, at positions 107 and 174 only MpAgo and the other two 5′-hydroxylated guide RNA binding Argonautes contain a charged residue (S7 Fig). We postulate that a combination of the unique orientation of the N-terminal domain and conserved DNA target-interacting residues results in a straight conformation of the heteroduplex for the 5′-hydroxylated guide RNA binding family of Ago proteins. In contrast, after two helical turns the directionality of the duplex held by RsAgo is diverted ~40° relative to the duplex of MpAgo (Fig 3C).

### MpAgo displays a symmetric tolerance for mismatches

**Fig 4:**
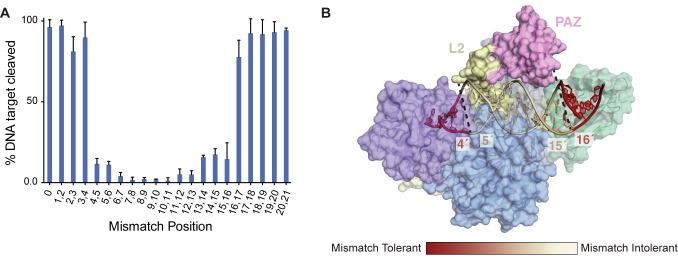
Symmetric mismatch tolerance across hybrid helix. (A) Dinucleotide mismatches were introduced across the entire guide RNA starting at the 5′ end. MpAgo cleavage kinetics of a ssDNA target was measured and the 30 min time point was plotted against mismatch position. Each reaction reached completion after 30 min. Error bars represent SD of three independent experiments. (B) Cartoon representation of the MpAgo RNA guide and DNA target heteroduplex. Heatmap coloring of the helix displays where dinucleotide mismatches inhibit DNA cleavage efficiency (white) or are tolerated (red).

Previous studies of MpAgo showed that single nucleotide mismatches between the guide and substrate strands at positions 5, 7, and 8 within the guide sequence reduced cleavage efficiency of a DNA target [12]. This suggested that the seed region of MpAgo may differ from the canonical seed region of other Ago proteins, nucleotides 2–8 of the guide strand [25]. To investigate this noncanonical seed region and mismatch tolerance, we introduced dinucleotide mismatches individually along the length of the guide RNA sequence (S1 Table). Dinucleotide mismatches up to positions 3 and 4 were tolerated with minimal to no decrease in cleavage efficiency, defined as the percent of DNA target cleaved after 30 minutes (Fig 4A). Introducing mismatches at positions 4 and 5 decreased cleavage efficiency to ~10% of that observed for a fully matched guide-substrate duplex, which gradually dropped to ~0% with mismatches introduced around the cleavage site, between positions 10 and 11 of the substrate DNA. The 3′ half of the guide strand displayed a symmetric mismatch tolerance profile similar to that of the 5′ half. Cleavage efficiency gradually increased to ~15% at positions 15–16, after which any dinucleotide mismatch did not significantly impact cleavage efficiency. The rate of target cleavage followed a similar symmetric profile, only differing with a decrease in cleavage rate with mismatches at positions 2–3 and 3–4 (S11 Fig and S2 Table).

Comparing MpAgo to the mismatch tolerance of TtAgo suggests that the straight orientation of MpAgo’s heteroduplex may play a role in the cleavage efficiency with mismatches [15]. Similar to MpAgo, single nucleotide mismatches around position 9–10 abolish cleavage efficiency of an RNA target. In contrast, mismatches after position 11 do not affect target cleavage. The guide-substrate duplexes of these two Ago complexes diverge structurally after position 11 of the TtAgo DNA guide, with MpAgo maintaining a linear conformation compared to the angled conformation observed for TtAgo (S8 Fig). Reduced cleavage efficiency occurs when dinucleotide mismatches are introduced at positions 5–15 of the MpAgo RNA guide. This region of the guide is positioned underneath Linker L2 and the PAZ domain, suggesting that Linker L2 and the PAZ domain assist in stabilizing heteroduplex in the appropriate position for cleavage (Fig 4B).

## Discussion

Although Argonaute proteins across all three domains of life share a conserved, four-domain architecture, their endogenous functions and mechanisms of action appear to differ. The crystallization of bacterial Argonautes TtAgo and RsAgo, and human Argonaute hAgo2, bound to their respective guide-substrate homo- or heteroduplexes have provided structural insights into preferential binding of guide and target strands. Here we present a structural analysis of a divergent bacterial Argonaute, MpAgo, bound to an RNA-DNA helix, revealing unique domain orientations and a distinct conformation of the helical substrate. These features extend the diversity of target recognition mechanisms observed for Argonaute proteins.

Argonaute proteins exhibit preferential binding to either RNA or DNA guide and substrate strands through various specific and non-specific interactions. Previous biochemical experiments showed that MpAgo binds RNA guides and preferentially targets DNA [12]. Analysis of the MpAgo ternary complex crystallized in this study revealed that the bound RNA-DNA heteroduplex adopts a B-form-like helical geometry. RNA-DNA hybrids naturally adopt an A-form geometry, which is less energetically stable than the A-form geometry adopted by RNA-RNA duplexes [33–35]. This implies that MpAgo deforms the RNA-DNA heteroduplex into a B-form-like geometry, and may explain why RNA targeting displays decreased cleavage efficiency relative to DNA targets. The target strand of the helix appears to interact with a positively charged cleft at the interface of the N and PAZ domains. Charged residues within this groove interact with the backbone of the DNA target, whereas an A-form-like helix may not be appropriately positioned for these stabilizing interactions. Although RsAgo also uses RNA guides to target DNA, the heteroduplex of the RsAgo ternary complex does not adopt a linear conformation. We speculate that this may be due to the position of the RsAgo N domain closer to the PIWI domain, which stabilizes a bent conformation of the heteroduplex. RsAgo may also not require the same mismatch tolerance as MpAgo, which we suggest is affected by the linear versus bent conformation of the duplex.

In addition to generating a charged cleft that stabilizes a DNA target strand, the positioning of the N domain close to the PAZ domain also induces a unique linear helical axis of the heteroduplex. In contrast, both TtAgo and RsAgo ternary complexes have N domains that are angled away from the PAZ domain. The helical substrates of these Argonautes bend after the second helical turn, which corresponds with the position of the N-terminal domains. In the case of TtAgo, the N domain acts as a wedge to block guide-target pairing and divert the target strand after position 11 [17]. This “passive” form of wedging has been proposed to correctly position target strands for cleavage, while an “active” form of wedging assists in separating miRNA duplexes bound to hAgo2 [32]. Similar to RsAgo, the N domain of MpAgo does not splay the heteroduplex, but instead stabilizes the helix through interactions with the target strand [31]. The absence of wedging by the MpAgo N domain may be necessary for the appropriate positioning of the target strand for cleavage. Alternatively, the endogenous guides of MpAgo may be loaded as single-stranded RNA (ssRNA) and thus wedging would not be necessary to actively unwind a duplex for passenger strand removal. When tiling dinucleotide mismatches along the length of the RNA-DNA hybrid helix, we observe that mismatches around the center of the guide RNA (positions 5 to 15) significantly inhibit cleavage efficiency, whereas the 5′ and 3′ ends display a strong tolerance for mismatches. The 3′ supplementary region (positions 13–16) of guide RNAs have been shown to be important for target recognition [36]. Our mismatch data confirm this observation, with mismatches in this region exhibiting decreased cleavage efficiency. In contrast, TtAgo displays a less symmetric mismatch tolerance profile, where mismatches within the 5′ region (positions 4 to 11) of the guide reduce cleavage efficiency and mismatches within the 3′ region (positions 13–19) show no effect [15]. The region where mismatches do not affect cleavage occurs within the bent portion of the helix. We hypothesize that the symmetric mismatch profile of MpAgo may be a result of the linear orientation of the heteroduplex, which places nucleotides 5 to 15 of the guide strand underneath the PAZ domain and Linker L2.

The target-bound MpAgo structure extends our current understanding of Argonaute diversity. This and related structures also highlight the differences between Argonaute proteins and the RNA-guided CRISPR-Cas (clustered regularly interspaced short palindromic repeats)-(CRISPR associated) effector proteins [37]. CRISPR-Cas enzymes have been widely adopted for applications involving RNA-guided nucleic acid recognition and cleavage [38], raising the possibility of similar biotechnological adaptation of Argonautes [39]. In contrast to CRISPR-Cas enzymes, however, Argonaute enzymes including MpAgo do not catalyze guide-directed dsDNA cleavage and they cannot unwind or displace a duplex substrate. Nonetheless, Argonaute proteins have the potential to be employed for ssDNA and ssRNA detection and cleavage, and may have different tolerance for guide strand length and mismatches to substrate strands based on available data. Additionally, despite a preference for DNA targeting, the ability to bind RNA targets may enable use of MpAgo and related enzymes for intracellular RNA-tracking and RNA pulldowns. The natural diversity of Argonaute proteins and their widespread occurrence across phylogeny implies adaptation for a variety of biological functions that have yet to be determined.

## Materials and methods

### Cloning and purification of MpAgo

The sequence encoding *M*. *piezophila* Argonaute (MpAgo) was codon-optimized for expression in *E*. *coli* and cloned into a custom pET-based expression vector using ligation independent cloning. The cloned construct encodes a fusion protein containing an N-terminal His_10_-tag followed by an Asn_10_-linker, Maltose Binding Protein (MBP), and a PreScission protease cleavage site. For crystallization, the D516A mutation for crystallography was introduced using QuikChange site-directed mutagenesis and verified by DNA sequencing.

The wildtype and mutant proteins were expressed in *E*. *coli* strain BL21(DE3) (New England Biolabs). For protein expression, cells were grown in TB medium to an OD_600_ of 0.8, expression was induced by addition of IPTG to 0.5 mM final concentration, and cells were incubated at 16°C while shaking for 16 h. The cell pellets were resuspended in 50 mM Tris-HCl pH 7.5, 300 mM NaCl, 1 mM TCEP, 0.5% (v/v) Triton-X 100, 10 mM imidazole, and supplemented with Complete protease inhibitor cocktail tablets (Roche). Cells were lysed via sonication and clarified lysate was bound in batch to Ni-NTA agarose (Qiagen). The resin was washed with 50 mM Tris-HCl pH 7.5, 300 mM NaCl, 1 mM TCEP, and 10 mM imidazole and bound protein was eluted in wash buffer containing 300 mM imidazole. The His_10_-MBP affinity tag was removed by cleavage with PreScission protease, while the protein was dialyzed overnight at 4°C against 50 mM Tris-HCl (pH 7.5), 300 mM NaCl, 1 mM TCEP, 5% (v/v) glycerol, and 10 mM imidazole. The cleaved MpAgo protein was separated from the fusion tag by ortho/reverse Ni-NTA. The protein was dialyzed into 50 mM Tris-HCl (pH 7.5), 150 mM NaCl, 1 mM TCEP, 5% (v/v) glycerol, applied to a 5 ml Heparin HiTrap column (GE Life Sciences), and eluted with a linear gradient of 0.15–1.2 M NaCl. Final purification was achieved by size exclusion chromatography using a HiLoad 16/60 Superdex 200 column (GE Life Sciences) in 50 mM Tris-HCl pH 7.5, 300 mM NaCl, 1 mM TCEP, and 5% (v/v) glycerol. Eluted protein was concentrated, flash-frozen in liquid nitrogen, and stored at −80°C.

### Oligonucleotide purification

All DNA and short RNA oligonucleotides were purchased from Integrated DNA Technologies (IDT). All synthetic oligonucleotides used for cleavage assays and crystallography experiments were gel-purified and quality-checked by Urea-PAGE prior to use

### In vitro cleavage assays

Purified oligonucleotide target DNA (10 pmol) was radiolabeled using T4 polynucleotide kinase (PNK) (NEB) and [γ-^32^P] ATP (Perkin Elmer) in 1× T4 PNK buffer (NEB) at 37°C for 30 min. The T4 PNK was heat inactivated at 65°C for 20 min. The labeling reactions were purified with illustra MicroSpin G-25 columns (GE Life Sciences). For single turnover experiments, MpAgo–RNA complexes were reconstituted by mixing 1 nM MpAgo with 1 nM guide strand in 10 mM Tris-HCl (pH 7.5), 150 mM NaCl, 2 mM MnSO_4_, 2 mM DTT, 5% (v/v) glycerol and incubating at 37°C for 30 min. Cleavage reactions were initiated by addition of 0.1 nM radiolabeled DNA or RNA substrates and performed at 60°C. 10 µl aliquots were removed at various time points and quenched by mixing with an equal volume of formamide gel loading buffer supplemented with 50 mM EDTA. Cleavage products were resolved by 12% (v/v) Urea-PAGE and visualized by phosphorimaging. Cleavage experiments were tested in three independent experiments. Percentage of cleavage was analyzed by densitometry using ImageQuant (GE Healthcare) and the average of three independent experiments was plotted against time (Prism).

### Crystallization and structure determination

Crystals were obtained by hanging-drop vapor diffusion at 18°C. Purified D516A MpAgo was incubated with a 1.2x molar amount of RNA on ice for 30 min. After incubation, DNA target was added at an equimolar amount to the RNA guide and the complex set up for crystallization. Original crystallization conditions were identified by sparse-matrix screen using 400 nl drops set over 70 μl reservoir solutions in a 96-well format (Falcon). Optimized crystals were grown in Easy-Xtal 15-well trays (QIAGEN) in 2 μl drops with a 1:1 ratio of protein and reservoir solution with a final protein concentration ~3.5 mg ml^-1^ in 0.1 M MES pH (7.0), 275 mM KI, and 27% (w/v) PEG 4000. Crystals were transferred to a cryoprotectant solution containing reservoir solution supplemented with 20% (v/v) ethylene glycol and incubated for approximately 30 s before flash freezing in liquid nitrogen. Data was collected under cryogenic conditions at the Lawrence Berkeley National Laboratory Advanced Light Source (Beamline 8.3.1).

X-ray diffraction data were processed with XDS and merged in AIMLESS [40] using the SSRL autoxds script (A. Gonzalez, SSRL). Indexed crystals belonged to the space group P 2_1_2_1_2_1_ with two copies of MpAgo in the asymmetric unit. The MpAgo-RNA complex (PDB ID: 5I4A) was used as a model for molecular replacement using the Phaser-MR program within PHENIX [41,42]. An initial electron density map was used for iterative building with Coot and refinement with PHENIX until all interpretable electron density was modeled [43].

## Supporting information

S1 Fig Electron density of RNA-DNA heteroduplex. The (2F_obs_−F_calc_) electron density map, contoured at 1σ, shows that 20 of the 21 nucleotides of the heteroduplex were resolved and modeled.
(EPS)

S2 Fig 3′ terminal base of DNA target is flipped away from RNA guide. (A) the 5′ terminal nucleotide of the guide strand does not base pair with the 3′ terminal nucleotide (C21) of the DNA target (red). F410 of the MID domain inserts between C20 and C21, which diverts the target away from the MID domain. K279 of Linker L2 interacts with the phosphate backbone to stabilize the kink. (B) Structure based sequence alignment of six Argonaute proteins using Promals3D. The top three Ago proteins preferentially bind 5′-hydroxylated guide RNAs. Residues are color-coded using the Clustal X color scheme. The aromatic residue inducing a kink at the 3′ end of the target is highlighted with a black dot.
(EPS)

S3 Fig Target binding leads to glutamate finger positioning towards active site. Comparing the guide-bound MpAgo complex (PDB ID: 5I4A, soft pink) to the target-bound complex (blue) reveals a conformation change in the PIWI domain. ß-strands 1 and 2 twist away from α-helix 1 in the direction of the black arrow. This movement creates space for the glutamate finger (E482) to enter the active site and complete the catalytic tetrad.
(EPS)

S4 Fig Charged cleft between the N-terminal and PAZ domains stabilize DNA target strand. Electrostatic surface potential of MpAgo generated in PyMol. The 5′ region of the DNA target (red) is stabilized by a positively charged cleft (blue) between the N-terminal and PAZ domains.
(EPS)

S5 Fig Heteroduplex bound to MpAgo adopts B-form-like helix. (A) Surface representation of MpAgo bound to guide RNA (orange) and target DNA (red) with only the backbones depicted. A 20 nt ideal A-form DNA helix (blue) was both generated and aligned via the guide strand in COOT. The black arrow indicates the displacement of the theoretical A-form target strand away from the charged interface between the PAZ and N domains. (B) Same representation as in (A) but with an ideal B-form DNA helix (green) aligned with the crystallized heteroduplex. The theoretical helix closely matches the observed heteroduplex.
(EPS)

S6 Fig N-terminal domain orientation stabilized by key residues. (A) The N-terminal domain (green) and Linker L1 (grey) of MpAgo are stabilized in close proximity by π -stacking of F109 and F96 (stick representation). Hydrogen bonding (black line) between N38 and D98 assist in the positioning of α-helix 1 relative to α-helix 3. (B) π -stacking of F55 to W85 (stick representation) of RsAgo (grey) helps stabilize the orientation of α-helix 1 relative to α-helix 3, resulting in the tilted direction of the N-terminal domain.
(EPS)

S7 Fig Conservation of aromatic and charged residues amongst Argonaute proteins. Structure based sequence alignment of six Argonaute proteins using Promals3D. The top three Ago proteins preferentially bind 5′-hydroxylated guide RNAs. Residues are color-coded using the Clustal X color scheme. Aromatic residues that position the N-terminal domain closer to the PAZ domain through π-stacking are highlighted with a black dot. Charged residues involved with DNA target interaction are highlighted with a red dot.
(EPS)

S8 Fig Helices bound to MpAgo and TtAgo adopt distinct trajectories due to different orientations of their respective N domains. (A) MpAgo (colored by domain) was aligned to TtAgo (PDB ID: 4NCB, light blue) using their PIWI domains. The MpAgo RNA guide (orange) and DNA target (red) heteroduplex remains in a linear conformation between to the two Ago lobes. After two helical turns, the RNA guide (green) and DNA target (green) of TtAgo angles behind the PIWI domain and away from the PAZ domain. (B) A cartoon representation of the linear heteroduplex of MpAgo (red) superimposed on the bent homoduplex of TtAgo (green). Divergence of the helices occurs after position 11 of the TtAgo DNA guide strand.
(EPS)

S9 Fig Charged residues of N-lobe interact with DNA target backbone. A close up view of the charged cleft formed at the intersection of the N (green) and PAZ (pink) domains, and Linker L1 (grey). Charged residues (K174, K93, N107) are visualized in stick representation, and interactions with the phosphate backbone of the DNA target (red) are indicated by a dotted black line.
(EPS)

S10 Fig MpAgo cleavage of ssDNA target using mismatched guide RNAs. Representative denaturing PAGE (one of three independent experiments) showing MpAgo cleavage kinetics. Radiolabeled ssDNA targets (50 nt) were incubated with MpAgo bound to an array of guide RNAs with dinucleotide mismatches from position 0–21. The labeled gel shows three different guides as examples. The final 30 min time point of each guide was quantified for comparison against all dinucleotide mismatch positions (Fig 3A).
(EPS)

S11 Fig Cleavage rate constants of MpAgo cleavage of ssDNA target using mismatched guide RNAs. Cleavage rate constants were calculated from time course cleavage assays (S10 Fig), using a nonlinear regression analysis. The plateau of the regression curves was set to 100% cleavage in order to calculate cleavage constants for mismatches with low or ablated cleavage efficiency. Three replicate experiments were performed and the rate constants are plotted against mismatch position.
(EPS)

S1 Table DNA and RNA oligonucleotides used and their sequences. (DOCX)

S2 Table Cleavage rate constants of MpAgo cleavage of ssDNA target using mismatched guide RNAs. (DOCX)
