# Supplementary material for: DNA recognition by an RNA-guided bacterial Argonaute
Source: PLoS One. 2017 May 17;12(5):e0177097. doi: 10.1371/journal.pone.0177097 (PMC5435312; doi:10.1371/journal.pone.0177097)
Supplement: S1 Table — (DOCX) [file pone.0177097.s012.docx]

| No. | Purpose | Sequence 5′-3′ |
| --- | --- | --- |
| 1 | 21 nt ssRNA guide for crystallography | GGUACAACCUACUACCUCAUU |
| 2 | 21 nt ssDNA target for crystallography | AATGAGGTAGTAGGTTGTACC |
| 3 | 50 nt ssDNA target for *in vitro* cleavage | GGACTATGATCTAGATATTAATGAGGTAGTAGGTTGTACCTCAGGAGGTC |
| 4 | 30 nt ssDNA marker | GGACTATGATCTAGATATTAATGAGGTAGT |
| 5 | 21 nt ssRNA mismatch at position 1,2 | **UU**UACAACCUACUACCUCAUU |
| 6 | 21 nt ssRNA mismatch at position 2,3 | G**UG**ACAACCUACUACCUCAUU |
| 7 | 21 nt ssRNA mismatch at position 3,4 | GG**GC**CAACCUACUACCUCAUU |
| 8 | 21 nt ssRNA mismatch at position 4,5 | GGU**CA**AACCUACUACCUCAUU |
| 9 | 21 nt ssRNA mismatch at position 5,6 | GGUA**AC**ACCUACUACCUCAUU |
| 10 | 21 nt ssRNA mismatch at position 6,7 | GGUAC**CC**CCUACUACCUCAUU |
| 11 | 21 nt ssRNA mismatch at position 7,8 | GGUACA**CA**CUACUACCUCAUU |
| 12 | 21 nt ssRNA mismatch at position 8,9 | GGUACAA**AA**UACUACCUCAUU |
| 13 | 21 nt ssRNA mismatch at position 9,10 | GGUACAAC**AG**ACUACCUCAUU |
| 14 | 21 nt ssRNA mismatch at position 10,11 | GGUACAACC**GC**CUACCUCAUU |
| 15 | 21 nt ssRNA mismatch at position 11,12 | GGUACAACCU**CA**UACCUCAUU |
| 16 | 21 nt ssRNA mismatch at position 12,13 | GGUACAACCUA**AG**ACCUCAUU |
| 17 | 21 nt ssRNA mismatch at position 13,14 | GGUACAACCUAC**GC**CCUCAUU |
| 18 | 21 nt ssRNA mismatch at position 14,15 | GGUACAACCUACU**CA**CUCAUU |
| 19 | 21 nt ssRNA mismatch at position 15,16 | GGUACAACCUACUA**AA**UCAUU |
| 20 | 21 nt ssRNA mismatch at position 16,17 | GGUACAACCUACUAC**AG**CAUU |
| 21 | 21 nt ssRNA mismatch at position 17,18 | GGUACAACCUACUACC**GA**AUU |
| 22 | 21 nt ssRNA mismatch at position 18,19 | GGUACAACCUACUACCU**AC**UU |
| 23 | 21 nt ssRNA mismatch at position 19,20 | GGUACAACCUACUACCUC**CG**U |
| 24 | 21 nt ssRNA mismatch at position 20,21 | GGUACAACCUACUACCUCA**GG** |
