# Supplementary material for: DNA recognition by an RNA-guided bacterial Argonaute
Source: PLoS One. 2017 May 17;12(5):e0177097. doi: 10.1371/journal.pone.0177097 (PMC5435312; doi:10.1371/journal.pone.0177097)
Supplement: S2 Table — (DOCX) [file pone.0177097.s013.docx]

| Mismatch Positions | Cleavage Rate Constant  (% Cleaved • min^-1^) |
| --- | --- |
| 0 | 45.8 ± 6.1 |
| 1,2 | 55.6 ± 5.1 |
| 2,3 | 13.2 ± 2.1 |
| 3,4 | 16.5 ± 2.5 |
| 4,5 | < 1.0 |
| 5,6 | < 1.0 |
| 6,7 | < 1.0 |
| 7,8 | < 1.0 |
| 8,9 | < 1.0 |
| 9,10 | < 1.0 |
| 10,11 | < 1.0 |
| 11,12 | < 1.0 |
| 12,13 | < 1.0 |
| 13,14 | < 1.0 |
| 14,15 | < 1.0 |
| 15,16 | < 1.0 |
| 16,17 | 7.7 ± 0.1 |
| 17,18 | 30.0 ± 4.6 |
| 18,19 | 41.5 ± 5.6 |
| 19,20 | 44.4 ± 5.4 |
| 20,21 | 48.6 ± 5.3 |
